# Pleomorphic Multinucleated Plasma Cells Simulating Megakaryocytes in an Anaplastic Variant of Myeloma

**DOI:** 10.4274/tjh.2017.0329

**Published:** 2018-05-25

**Authors:** Shivangi Harankhedkar, Ruchi Gupta, Khaliqur Rahman

**Affiliations:** 1 Sanjay Gandhi Post Graduate Institute of Medical Sciences, Department of Hematology, Lucknow, Uttar Pradesh, India

**Keywords:** Myeloma, Anaplastic, Megakaryocytes

To the Editor,

Myeloma cells are notorious for their morphological variations, which range from mature-appearing plasma cells to other poorly differentiated forms. The pleomorphic or anaplastic variants are its uncommon rare variants, which may pose a diagnostic dilemma in unprecedented cases. These anaplastic variants may mimic high-grade lymphomas, leukemia, or even metastatic carcinomas [1,2]. Anaplastic plasma cells may be seen at diagnosis or evolve during the terminal phase of the disease [3]. The correlation of this morphological variant with treatment outcome is controversial, but it is believed to be a harbinger of aggressive disease [4,5]. Herein we report the case of an unsuspected multiple myeloma, where bone marrow examination revealed the presence of bizarre plasma cells simulating megakaryocytes. 

An asymptomatic 65-year-old diabetic male presented with bicytopenia. Complete blood count analysis showed hemoglobin of 7 g/dL, total leukocyte count of 6.3x10^9^/L, and 51x10^9^/L platelets. The peripheral smear showed the presence of occasional circulating plasma cells with minimal rouleaux formation. Bone marrow examination revealed proliferation of highly pleomorphic cells with multinucleation, simulating megakaryocytes. Cells had moderate to abundant basophilic cytoplasm, while nuclei were multilobulated, with open chromatin and prominent nucleoli, along with a few intranuclear basophilic inclusions (Figure 1A). Serum protein electrophoresis revealed monoclonal protein of 0.19 g/dL, which was confirmed to be IgA kappa on immunofixation (Figure 1B). The kappa/lambda ratio was 427.6 and the β2 microglobulin level was 21.9 mg/L. Immunophenotypically, the cells expressed CD38, CD138, CD56, and CD200 (Figure 1C-1E). FISH analysis, performed after magnetic bead enrichment of plasma cells, showed the presence of del(13q14.3). The patient was unfortunately lost to follow-up.

Anaplastic multiple myeloma (AMM) is a rare morphological variant of multiple myeloma, the true incidence of which is largely unknown [1,2,6,7]. In the early 1990s, Allen and Coleman [3] reviewed 108 cases of anaplastic myeloma, 68 of which showed the presence of extramedullary disease. Other salient characteristics of AMM, which have been observed by other authors, too, include a younger age atpresentation, cytopenias, predilection for IgA myelomas, and aggressive clinical course [4,7,8,9]. Bahmanyar et al. [10] reviewed the genetic features of 11 cases of AMM for the presence of myeloma-associated genetic abnormalities and compared them with 188 newly diagnosed non-anaplastic variants of MM. They observed significantly higher frequencies of 1q21 amplification, 17p(p53) deletion, and t(4, 14). Additionally, the presence of complex karyotype, del(13q14.3), t(1;19), and near tetraploidy has also been reported [8,9,10]. The treatment outcome of this variant is considered poor as per the older literature; however, patients treated with triple-drug chemotherapeutic regimens in the modern era have shown sustained responses [1,5,9].

To conclude, awareness of these variants in myeloma is important for an accurate diagnosis. In cases where myeloma cells show extreme “de-differentiation”, a multidisciplinary approach with the addition of immunophenotyping in the diagnostic armamentarium is recommended. With the advent of triple-drug regimens in myeloma therapy and autologous bone marrow transplantation, the outcome of this variant needs to be re-addressed inlarger studies.

## Figures and Tables

**Figure 1:**
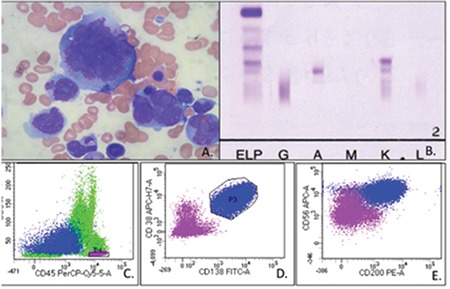
Panel of photomicrographs: A) May-Grünwald Giemsa stained bone marrow aspirate smear (100^x^) showing pleomorphic cells, with multilobation and multinuclearity, with prominent inclusions (red arrows) and abundant basophilic cytoplasm, and absence of perinuclear hof; B) serum immunofixation highlighting presence of IgA kappa monoclonal protein; C, D, E) panel of dot plots documenting these atypical plasma cells to be positive for CD38, CD138, CD200, and CD56 and negative for CD45.
